# The Association between Polymorphisms in the MRPL4 and TNF-α Genes and Susceptibility to Allergic Rhinitis

**DOI:** 10.1371/journal.pone.0057981

**Published:** 2013-03-05

**Authors:** Xin Wei, Yuan Zhang, Zheng Fu, Luo Zhang

**Affiliations:** 1 Department of Otolaryngology, Head and Neck Surgery, Beijing Tongren Hospital, Capital Medical University, Beijing, PR China; 2 Key Laboratory of Otolaryngology, Head and Neck Surgery (Ministry of Education of China), Beijing Institute of Otorhinolaryngology, Beijing, PR China; 3 Department of Otolaryngology, Head and Neck Surgery, People’s Hospital of Hainan Province, Haikou, PR China; Yale School of Public Health, United States of America

## Abstract

**Background:**

Allergic rhinitis (AR) is a chronic inflammatory disease of the nasal mucosa, involving a complex interaction between genetic and environmental factors. Evidence suggests that polymorphisms in the gene coding for mitochondrial ribosomal protein L4 (MRPL4), located in close proximity to intercellular adhesion molecule-1 (ICAM-1) gene on chromosome location 19p13.2, may influence the risk factor for the development of AR.

**Objective:**

The aim of our study was to investigate any association between AR susceptibility and polymorphisms in ICAM-1 gene, as well as associations between AR risk and polymorphisms in MRPL4, nuclear factor-kappaB (NF-κB) and tumor necrosis factor alpha(TNF-α) genes, associated with ICAM-1 expression.

**Methods:**

A cohort of 414 patients with AR and 293 healthy controls was enrolled from the Han Chinese population in Beijing, China. Blood was drawn for DNA extraction and total serum immunoglobulin E (IgE). A total of 14 single nucleotide polymorphisms (SNPs) in ICAM-1, NF-κB, TNF-α, and MRPL4 genes were selected using the CHB genotyping data from the International Haplotype Mapping (HapMap) and assessed for differences in frequencies of the alleles and genotypes between the AR patients and control subjects.

**Results:**

TNF-α SNP rs1799964 and MRPL4 SNP rs11668618 were found to occur in significantly greater frequencies in the AR group compared to control group. There were no significant associations between SNPs in NF-κB, ICAM-1 and AR. The SNP-SNP interaction information analysis further indicated that there were no synergistic effects among the selected sets of polymorphisms.

**Conclusions:**

Our results suggest a strong association between AR risk and polymorphisms of MRPL4 and TNF-α genes in Han Chinese population.

## Introduction

Allergic rhinitis (AR) is a chronic inflammatory disease of the nasal mucosa, which is caused by a complex interaction between genetic and environmental factors involving allergen-IgE-mediated stimulation of a variety of immune competent cells and release or expression of a variety of proinflammatory cytokines and mediators in allergen-sensitized individuals. AR morbidity varies from10%to25% in different regions and countries; with around 11. 1% of the population in China being affected according to a telephone questionnaire survey [1].

A first genome-wide association study for atopy and AR conducted in a cohort of Chinese volunteers in Singapore has recently indicated that there may be an association between susceptibility to atopy and/or AR and polymorphisms in two candidate genes; i) the *MRPL4* gene coding for the 39S mitochondrial ribosomal protein L4 and ii) the *BCAP* gene coding for the cytosolic B-cell adapter for phosphatidylinositol 3-kinase (BCAP) [2].In this respect the MRPL4 gene is of particular interest as it is locatedonchromosomelocation19p13.2, with the gene coding for intercellular adhesion molecule-1 (ICAM-1),which plays a key role in allergic disease [3], [4].One study investigated the relationship between the severity of disease and the levels of serum soluble ICAM-1 (sICAM-1) and expression of ICAM-1 in nasal mucosa of patients with persistent allergic rhinitis, and demonstrated that both serum sICAM-1 levels and nasal ICAM-1 expression were significantly higher in patients with moderate-to-severe rhinitis, compared to patients with mild rhinitis [5]. A more recent study by Li and colleagues has demonstrated that nasal saline irrigation as an adjunct treatment to tapered topical steroid treatment after 8 and 12 weeks in children with AR resulted in significantly decreased concentrations of sICAM-1 in nasal secretions accompanied by significant improvements in symptoms with lower concentrations of topical steroid.

Despite the well-documented role of ICAM-1 in AR, the mechanism/s underlying the regulation of ICAM-1 in allergic diseases is still not clear. Zhao and colleagues have shown that the expression of both the transcription factor nuclear factor-kappaB (NF-κB) and ICAM-1wassignificantly higher in patients with AR compared with non-rhinitic control subjects and that the expression of ICAM-1 mRNA was significantly correlated with the expression of NF-κB [6]. Another study has demonstrated that tumour necrosis factor alpha(TNF-α)significantly increased the expression of ICAM-1 gene expression in cultured human nasal epithelia cells *in vitro* and that the TNF-mediated-ICAM-1 mRNA and ICAM-1 surface expression at 48 h was significantly inhibited by incubation with human recombinant soluble TNF receptor I and dexamethasone [7]. To our knowledge, the association between ICAM-1 gene polymorphisms and predisposition of an individual to AR has not been reported to date. The aim of our study was therefore to investigate any associations between polymorphisms in ICAM-1 gene, in a Han Chinese cohort. Furthermore, we aimed to investigate associations between polymorphisms in MRPL4, NF-κB and TNF-α gene, which may influence ICAM-1 expression, and AR susceptibility in this cohort.

## Materials and Methods

### Study Subjects

414 outpatients (239 males and 175 females) with AR were recruited prospectively from the Otorhinolaryngology department of the people's hospital of Hainan province from February 2010 to February 2011. 293 healthy volunteers with ethnically similar background were also recruited from the local population as controls to determine background allele frequencies in the population.

Patients with a documented history of AR were included if they fulfilled all the criteria of the Allergic Rhinitis and its Impact on Asthma(ARIA) guidelines [8], including 1) demonstration of persistent or discontinuous symptoms of anterior rhinorrhoea, continuous sneezing, nasal obstruction and itching, 2) demonstration by nasal endoscopy of a pale and oedematous nasal mucosa, nasal discharge and swollen inferior turbinates and 3) positive serum antigen-specific IgE, as measured by the ImmunoCAP 100 system (Pharmacia, Uppsala, Sweden) or positive antigen skin prick test (SPT) (Allergopharma, Reinbeck, Germany). A diagnosis of AR was additionally confirmed by a positive skin test response to the sensitising allergen and the presence of symptoms following exposure to the same allergen. Diagnosed AR individuals were excluded from the study if they suffered from (1) hypertension, diabetes or any other chronic disease; (2) tumor in the nasal cavity; or (3) eczema or asthma.

The antigens employed for allergy tests included house dust mite (HDM) (Der f and Der p); seasonal grass pollens (Gaint Ragweed; Mugwort; Lamb’s quarers; Humulus; Chenopodium album and so on); animal Hair (especially dog and cat); moulds (indoor and outdoor mustiness or floricultural environment) and cockroach. Subjects were considered to be sensitized to allergens when serum IgE was ≥0.35 kU/l. A positive SPT result was defined as a wheal size greater than or equal to one half of the diameter of the histamine control and at least 3 mm larger than the diameter of the negative control [9]. Individuals showing positive results to two or more allergens in the skin test or serum examination were defined as being poly sensitised. Serological and skin testing were performed by specialist technicians and nurses respectively, while the AR diagnoses were made by clinical rhinologists.

The healthy controls presented no clinical features, local nasal cavity signs, or a family history of allergic disease, and showed negative results for serum antigen-specific IgE.

All the subjects were of Han Chinese ethnic origin from the southern region of China and provided written informed consent prior to entry in the study. The study protocol was approved by the Ethics Committee of the People's hospital of Hainan province and performed in accordance with the guidelines of the World Medical Association's Declaration of Helsinki.

### Selection of Polymorphisms in the humanicam-1, NF-κB, TNF-α and MRPL4 Genes

The majority of the tag SNPs (tSNPs) were chosen from the Hapmap database according to the following selection strategy: Firstly, the International Haplotype Mapping (HapMap) (www.hapmap.org) SNP databases were used to select tSNPs in the ICAM-1, NF-κB, TNF-α and MRPL4 genes region, and the screened region extended 10 kilobases upstream of the annotated transcription start site and downstream at the end of the last each gene exon. The tSNPs were selected to extract most of the genetic information in the region using the CHB genotyping data from the HapMap database (HapMap data rel 27 Phase II +III, Feb2009) [10]. From this dataset, genotyping data for 20 tSNPs were obtained and loaded in the Haploview software version 4.1 [11]. Secondly, tSNPs were then selected using a pairwise tagging algorithm setting the Hardy-Weinberg *p* value, minor allele frequency (MAF) and r2 thresholds at 0.01, 0.05 and 0.8, respectively. The linkage disequilibrium (LD) pattern of each gene in the CHB population exhibited strong LD in several groups of tSNPs (r2≥0.8), indicating that most common SNPs can be captured by a subset of tagging SNPs. Consequently, we choose 14 SNPs, including rs5498, rs281432, rs281428, rs1059840, rs8104608, rs11668618, rs13117745, rs230530, rs3774963, rs1598861, rs118882, rs1800629, rs3093668, and rs1799964 to represent the four genes loci for eventual genotyping.

Single nucleotide polymorphism genotyping DNA was isolated from peripheral blood leukocytes and collected in EDTA-treated tubes, using commercial DNA Isolation Kits for Mammalian Blood (Roche, Indianapolis, USA). Isolated DNA from the blood was stored at 4°C and used in further investigations within 2 days of isolation. The majority of the selected SNP genotyping was performed with the Sequenom MassARRAYiPLEX Gold platform (Sequenom, San Diego, California) according to the manufacturer’s instructions. The polymerase chain reaction (PCR) and extension primers were designed using MassARRAY Assay Design 3.1 software (Table 1). Genotyping was performed without knowledge of the case or control status. A 10% random sample was tested in duplicate by different investigators to test the reproducibility of the assay; which was shown to be 100%.

**Table 1:** Primers used in the screening of SNPs by MassArray.

Gene & position	SNP	Alleles	Primers	Extension Primers
MRPL4 (Chromosome: 19; Location: 19p13.2)	rs1059840	A/T	ACGTTGGATGAGCGGCCACCCCGTACCAC	CCCCGTACCACTGTTGA
			ACGTTGGATGAGGCTCCCAAGTCGGCCAG	
	rs8104608	A/G	ACGTTGGATGTCCTTGGCCAGAGGCTTACA	cctCTTACAGGCAACAAGC
			ACGTTGGATGTAGTGTGACTTTGTGAGCCC	
	rs11668618	C/T	ACGTTGGATGGACAAAACAGGCAAGGTCCG	ggttTGCAAGTGGCTCAGG
			ACGTTGGATGACCCACCTCAGCTTCCAGT	
ICAM-1 (Chromosome: 19; Location: 19p13.3–p13.2)	rs5498	A/G	ACGTTGGATGACTCACAGAGCACATTCACG	CATTCACGGTCACCT
			ACGTTGGATGTTGAGGGCACCTACCTCTGT	
	rs281432	C/G	ACGTTGGATGAGCTGGGACTTTCCTTCTTG	GAGTCATGGAGGGTTT
			ACGTTGGATGGCCTTCAGCTATCTAATCCC	
	rs281428	C/T	ACGTTGGATGTGGAATTACAGGCGCCCAGCA	ccccaGCCCAGCACCACGCC
			ACGTTGGATGGCCAACATGATGAAATCCCG	
NF-κB (Chromosome: 4; Location: 4q24)	rs13117745	C/T	ACGTTGGATGGACTACCCCTGATAAAGTCC	GGGGTAGTTGTTCCTATC
			ACGTTGGATGAATGAGAGGGCCCTTCTTTG	
	rs230530	C/T	ACGTTGGATGGGACATACAAGCATTCTCC	TAGCACCAAACATCTTAATTT
			ACGTTGGATGGGCATATGGTGGTTCTCATT	
	rs3774963	C/G	ACGTTGGATGATGGAAGGCATGGTGTTTGG	gggATGGTGTTTGGAATGTTC
			ACGTTGGATGGTCTATGACTGTTTCAGACT	
	rs1598861	A/C	ACGTTGGATGTTGTACCAAAACCCTGCTTC	ATGAATCCATTTAATGAAGTTTAT
			ACGTTGGATGATCCTTTTCGACTAGCCTAC	
	rs118882	A/G	ACGTTGGATGCTCTCACAAAGGCATGACAG	agggcGACAGGGTGTGAGGTTGGA
			ACGTTGGATGCTGTGCTTTTCCTCAGCTAC	
TNF-α (Chromosome: 6; Location: 6p21.3)	rs1800629	A/G	ACGTTGGATGGATTTGTGTGTAGGACCCTG	cagcGGCTGAACCCCGTCC
			ACGTTGGATGGGTCCCCAAAAGAAATGGAG	
	rs3093668	C/G	ACGTTGGATGAGGTTGCAGAGTTAGGACAG	ggggaTGATTTGAAGCCTAGACA
			ACGTTGGATGACCTGAATCACACAGCCAAG	
	rs1799964	C/T	ACGTTGGATGGGGAAGCAAAGGAGAAGCTG	cacGCAAAGGAGAAGCTGAGAAGA
			ACGTTGGATGTGTAACCCATTCCTCAGAGC	

### Statistical Analyses

Data were initially processed for suitability for further statistical evaluation using the Haploview version 4.1 software. Hardy-Weinberg equilibrium (HWE) of each SNP was assessed in controls only and a threshold P<0.05 was regarded to indicate deviation from HWE. In addition, we assessed the MAF, non-missing genotype percentage and other criteria in the AR cases as well as controls to filter the data. Among them, minor allele frequency (MAF) and non-missing genotype percentage thresholds were set at <0.001 and <95%, respectively.

Differences in frequencies of the alleles and genotypes between the AR subjects and control subjects were evaluated using the chi-square test and a P-value of 0.05 was considered significant. A correction of allele distribution χ^2^ test was made by using 100,000 permutation testing. The Global P values (2 degrees of freedom) were also calculated when we compared the genotype frequencies between cases and controls using a χ^2^ test. Akaike’s information criteria (AIC) were used to select the most parsimonious genetic model for each SNP. Odds ratios (ORs) and 95% confidence intervals (CIs) were calculated by unconditional logistic regression analysis, adjusted for age and gender. Moreover, tests for trend were done by including genotypes as an ordinal variable in regression models (df = 1) to obtain P values for trend (two-sided). These analyses were conducted using the STATA statistical package (version 11.0; Stata Corp LP, College Station, TX, USA).

A multistage strategy was employed for analysing gene–gene interactions. Firstly we first used the multifactor dimensionality reduction (MDR) method to detect and characterize locus–locus and gene–gene interaction models [12]. Interaction dendrograms and graphs based on entropy (measurement of randomness) estimates were subsequently employed to confirm, visualize, and interpret the interactions models identified by MDR.

The MDR approach [12], [13], [14]applies a constructive induction algorithm that creates a new attribute by pooling genotypes from multiple SNPs. This method includes a combined cross-validation/permutation-testing procedure that minimizes false-positive results by multiple examinations of the data. Models that are true-positives are likely to be generalized to independent datasets and will have estimated testing accuracies of greater than 0.5. In addition to the testing accuracy, we also employed the cross validation consistency (CVC), a measure of how many times out of 10 divisions of the data that MDR found the same best model. Among this set of best multifactor models, the combination of genetic factors that maximized the testing accuracy and/or the highest CVC was selected and further evaluated using permutation testing. Moreover, age and gender were included as environmental factors for MDR analysis. The MDR analysis was performed by using version 1.0.0 of the open-source MDR software package freely available online (http://www.multifactordimensionalityreduction.org/).

The statistical power for the study was calculated using G*Power 2 software (http://www.psycho.uni-duesseldorf.de/aap/projects/gpower/).

## Results

### Population Characteristics

The characteristics of the study population are shown in Table 2. The cohort of 414 AR patients had a mean age of 23 years and consisted of 57.7% male and 42.3% female patients. The control group comprised of similar proportions of male (59.7%) and female (40.3%) subjects, although the mean age of the group was higher (37 years) compared to the AR group. The mean total serum IgE level for the case and control groups were 312.2±560.4 and 74.8±152.5 IU/ml respectively.

**Table 2:** Demographic characteristics of the study population.

Characteristic	AR cases (n = 414)	Controls (n = 293)
Age Mean (Range) (years)	23.8±14.8 (4–81)	37.1±15.8 (2–81)
Sex, M/F, No. (%)	239 (57.7)/175 (42.3)	175 (59.7)/118 (40.3)
Total IgE, kU/l	312.2±560.4	74.8±152.5

The statistical power of AR vs. control was 95.01%, with the sample size of 414 AR patients and 293 controls; with α = 0.05 and the β = 0.2.

### Individual SNP Association Analysis

The initial quality tests for the SNPs in the four genes selected for genotyping demonstrated that SNP (rs281428) in ICAM-1 gene and SNP (rs11882) in NF-κB gene were not suitable for study, as indicated by significant deviation from the Hardy–Weinberg equilibrium (HWE) threshold of P<0.05 (P = 0.000 and 0.005 respectively). The data for these loci were therefore excluded from further analyses, and overall a total of 12 SNPs were chosen as shown in Tables 3 and 4.

**Table 3:** Allele frequencies and AR susceptibility.

Gene	SNP	Minor Allele	Case, Control Frequencies	P	OR (95% CI)	P^a^	OR (95% CI) a
MRPL4	rs1059840	A	0.106, 0.090	0.328	1.196 (0.836–1.712)	0.668	1.087 (0.741–1.596)
	rs8104608	A	0.117,0.122	0.794	0.957 (0.687–1.333)	0.500	0.884 (0.618–1.265)
	rs11668618	T	0.046, 0.069	0.065	1.534 (0.971–2.424)	**0.029**	**0.583 (0.358–0.947)**
ICAM-1	rs5498	G	0.232, 0.223	0.703	0.952 (0.738–1.228)	0.756	1.045 (0.794–1.375)
	rs281432	G	0.252, 0.269	0.468	1.094 (0.858–1.394)	0.386	0.891 (0.686–1.157)
NF-κB	rs13117745	T	0.064, 0.055	0.483	0.858 (0.541–1.337)	0.540	1.162 (0.719–1.879)
	rs230530	C	0.444, 0.454	0.722	0.962 (0.777–1.192)	0.649	0.948 (0.753–1.193)
	rs3774963	C	0.419, 0.444	0.350	0.902 (0.728–1.119)	0.483	0.920 (0.730–1.161)
	rs1598861	C	0.107, 0.103	0.775	0.951 (0.673–1.344)	0.808	1.047 (0.722–1.519)
TNF-α	rs1800629	A	0.107, 0.111	0.838	0.965 (0.688–1.355)	0.541	0.894 (0.623–1.282)
	rs3093668	C	0.021, 0.019	0.815	1.096 (0.509–2.357)	0.432	1.394 (0.609–3.190)
	rs1799964	C	0.230, 0.176	**0.014**	**1.399 (1.069–1.831)**	**0.049**	**1.337 (1.001–1.787)**

a Results from unconditional logistic regression analyses, adjusted for age and gender.

**Table 4:** Genotype frequencies and AR susceptibility.

Gene	SNP	Genotype	P	Global P^a^	OR (95% CI)	P^b^	OR (95% CI)	P^c^
MRPL4	rs1059840	AA	0.784	0.607	1.224 (0.290–5.169)	0.955	0.957 (0.207–4.413)	0.391
		AT	0.331		1.218 (0.818–1.814)	0.689	1.093 (0.708–1.686)	
	rs8104608	AA	0.747	0.945	0.724 (0.101–5.177)	0.842	0.813 (0.106–6.247)	0.793
		AG	0.847		0.965 (0.671–1.388)	0.655	0.913 (0.614–1.359)	
	rs11668618	CT	0.11	0.184	0.671 (0.412–1.094)	**0.034**	**0.564 (0.332–0.957)**	0.068
		TT	0.374		0.336 (0.030–3.72)	0.393	0.333 (0.027–4.150)	
ICAM-1	rs5498	AG	0.967	0.855	1.007 (0.730–1.389)	0.960	1.009 (0.709–1.437)	0.705
		GG	0.578		1.215 (0.611–2.417)	0.667	1.176 (0.562–2.463)	
	rs281432	CG	0.487	0.752	0.893 (0.649–1.228)	0.781	0.951 (0.670–1.351)	0.508
		GG	0.663		0.877 (0.486–1.582)	0.394	0.759 (0.403–1.430)	
NF-κB	rs13117745	CT	0.273	0.367	1.313 (0.806–2.139)	0.283	1.336 (0.787–2.267)	0.456
		TT	0.406		0.360 (0.033–3.997)	0.218	0.211 (0.018–2.503)	
	rs230530	CC	0.648	0.747	0.903 (0.584–1.397)	0.434	0.826 (0.512–1.333)	0.789
		CT	0.763		1.055 (0.746–1.492)	0.927	1.018 (0.697–1.485)	
	rs3774963	CC	0.234	0.185	0.772 (0.508–1.182)	0.490	0.848 (0.532–1.354)	0.359
		CG	0.496		1.126 (0.800–1.586)	0.510	1.135 (0.779–1.652)	
	rs1598861	AC	0.667	0.857	1.086 (0.745–1.583)	0.689	1.088 (0.719–1.646)	0.612
		CC	0.739		0.716 (0.100–5.117)	0.641	0.657 (0.112–3.842)	
TNF-α	rs1800629	AA	0.361	0.522	2.784 (0.309–25.072)	0.857	1.182 (0.193–7.233)	0.756
		AG	0.556		0.895 (0.618–1.296)	0.373	0.833 (0.557–1.246)	
	rs3093668	CG	0.813	0.813	1.098(0.506–2.380)	0.340	1.510 (0.648–3.519)	0.600
		CC	NA		NA	NA	NA	
	rs1799964	CC	0.823	**0.009**	1.106 (0.457–2.680)	0.840	0.911 (0.369–2.251)	**0.011**
		CT	**0.002**		**1.660(1.199–2.296)**	**0.005**	**1.664 (1.163–2.382)**	

a Global P values [2 degrees of freedom (df)]: genotype frequencies in cases and controls were compared using a χ^2^ test with 2 df.

b Results from unconditional logistic regression analyses, adjusted for age and gender.

c P values for trend (two-sided) derived from trend tests (df = 1).

NA: not available because of the rarity of genotype.

In the single-locus analyses of allele frequencies associated with increased AR risk, C allele of rs1799964 in TNF-α gene was found to be significantly different between the AR and control subjects (P = 0.014; OR = 1.399) (Table 3). Moreover, age and gender adjusted logistic regression analyses, as assessed by the Akaike’s information criteria (AIC) in the codominant effect model, further revealed that T allele of rs11668618 in MRPL4 gene (P = 0.029; OR = 0.583) was associated with decreased risk for AR, whereas the C allele of rs1799964 in TNF-α gene (P = 0.049; OR = 1.337) was associated with increased risk for the development of AR (Table 3). Furthermore, rs11668618 and rs1799964 remained significantly associated with AR after 100,000 permutations (P<0.05).

The genotype distributions of the 12 selected SNPs in AR and control subjects are summarized in Table 4. Again, rs1799964 in TNF-α gene (CT genotype, P = 0.002; OR = 1.660) was found to be significantly associated with AR susceptibility in these analyses. Age and gender adjusted logistic regression analyses again revealed that in the codominant-effect model rs11668618 in MRPL4 gene (CT genotype, P = 0.034; OR = 0.564) and rs1799964 in TNF-α gene (CT genotype, P = 0.0059; OR = 1.664) were associated with decreased and increased risk for development of AR, respectively. The rs1799964 showed significance levels (P_trend_ = 0.011) of the increasing trend were apparent for an indication of a dose-response relationship between the polymorphism and AR risk.

### Locus–locus and Gene–gene Interactions

None of the 12SNPs in the four genes subjected to SNP-SNP synergistic analysis using the MDR function, showed any synergistic effects (Table 5).

**Table 5:** MDR analysis summary.

Best candidate model	Training accuracy (%)	Permutation P-value	Cross-validation Consistency
rs1799964	56.00%	0.4793	10/10
rs13117745 rs1799964	57.84%	0.5155	5/10
rs281432 rs8104608 rs3774963	60.07%	0.4678	4/10
rs5498 rs281432 rs230530 rs1799964	63.11%	0.488	5/10

Table 6 summarizes the best interaction models obtained for the 12 tSNPs in MRPL4, ICAM-1, NF-κB and TNF-α in the MDR analysis adjusted for age and gender as variables. The model with the lowest prediction error and the highest CVC was selected and further evaluated using the permutation test. In the one-factor model, age was the best attribute for predicting AR risk (testing accuracy = 72.57%; CVC = 10; P = 0.001). The four-factor model (i.e., age, ICAM-1 SNP2 rs281432: C/G, NF-κB SNP3 rs3774963: C/G and TNF-α SNP3 rs1799964: C/T) was the best interaction model with the highest testing accuracy of90.83% (CVC = 9; P = 0.033). Although, the two-factor model (i.e., age and NF-κB SNP3 rs3774963: C/G) and the three-factor model (i.e., age, ICAM-1 SNP2 rs281432: C/G and NF-κB SNP3 rs3774963: C/G) also demonstrated significant interactions (P = 0.005 and 0.003, respectively), these models had lower testing accuracies (78.37% and 85.06%, respectively) than the four-factor model (Table 6).

**Table 6:** MDR analysis summary (including age).

Best candidate model	Training accuracy (%)	P-value^a^	Cross-validation Consistency
age	72.57	**0.001**	10/10
age rs3774963	78.37	**0.005**	10/10
age rs281432 rs3774963	85.06	**0.003**	7/10
age rs281432 rs3774963 rs1799964	90.83	**0.033**	9/10

a P values based on 1000 permutation test.

**Figure 1:**
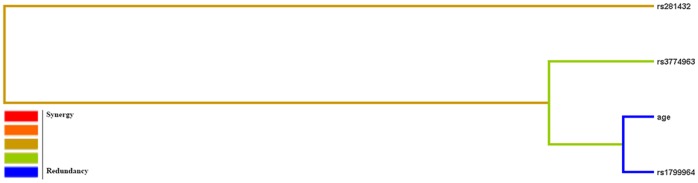
Dendrogram of SNP-SNP synergistic analysis by MDR. The dendrogram comprises a spectrum of colours representing a continuum from Synergy to Redundancy; with orange representing a relatively high degree of synergy (positive information gain) and blue representing redundancy (negative information gain).

Although the MDR analysis indicated the four-factor model to be the best model, it did not specify the presence of any synergistic relationships between the factors assessed. However, application of an interaction dendrogram and graph indicated that although the hierarchical cluster analysis placed age, NF-κB SNP3 and TNF-α SNP3 on the same branch (Figure 1), the blue line in the diagram suggested that the three-locus model may have a redundancy interaction effect on modulating risk of AR. ICAM-1 SNP2 was on another branch, revealing an interaction between them.

## Discussion

Although the pathogenesis of allergic rhinitis is still not entirely clear, there is general agreement about a chronic inflammatory response of nasal mucosa dominated by Th2 immune response and characterized by migration and infiltration of a large number of eosinophils and mast cells in the lesions of the nasal mucosa; with cell adhesion molecules playing a critical role in this process. In this regardICAM-1, a member of the immunoglobulin superfamily of adhesion molecules, has been shown to be particularly important in the pathogenesis of AR because it binds to lymphocyte function-associated antigen-1 (LFA-1), aβ2 integrin ligand located on a variety of pro-inflammatory cells, which infiltrate the nasal mucosa in AR [3], [4], [15], [16], [17], [18].

Similarly, nuclear factor-kappaB (NF-κB) has been shown to be important because it regulates genes of several pro-inflammatory mediators, including cytokines, chemokines, adhesion molecules, iNOS and costimulatory molecules [19], [20].In the case of the ICAM-I gene located in chromosome 19, the promoter site of this gene contains binding sites for NF-κB [21]. It has been demonstrated that on activation NF-κB enters into the nucleus and combines with its lCB motif, resulting in the increased ICAM-1 mRNA transcription [22]. ICAM-1 in turn further activates NF-κB, thus creating a positive feedback loop to amplify inflammation [23]. Despite the well-documented role of ICAM-1 and NF-κB in the pathogenesis of AR, to our knowledge, this study is the first to investigate the influence of genetic polymorphisms in the genes for these mediators and to demonstrate that there does not appear to be any direct associations between the selected polymorphisms in ICAM-1 (SNP rs281428) and NF-κB (SNP rs11882) genes and susceptibility to AR. These findings, however, do not preclude the possibility that other hitherto undetected SNPs in these genes might be associated with susceptibility to AR.

Studies have shown that NF-κB also regulates the expression of TNF-α gene [24], which is of particular significance because TNF-α has also been shown to be an important mediator in the induction and maintenance of inflammation in allergic rhinitis [5], [25], [26]. Present study has demonstrated that, unlike ICAM-1 and NF-κB polymorphisms, a locus (rs1799964) in TNF-α gene was significantly different in the AR and healthy people, suggesting the this particular SNP polymorphism of the TNF-α gene may be associated with the risk of AR in the Han Chinese population. Moreover, we meanwhile demonstrated a genotype-dependant association pattern regarding to rs1799964_TNF-α vs AR development, i.e. only the heterozygous (CT) but not homozygous genotypes of was significantly. Although, our findings for the TNF-α gene polymorphism are contrary to those of Zhu and colleagues [27], who did not find an association between the TNF-α allele and AR, they are nevertheless in accordance with the findings of several other studies. Gentile and co-workers [28] investigated genotypes for a number of different cytokines; including TNF-α, interferon gamma (IFN-gamma), interleukin (IL)-6, IL-10, and transforming growth factor (TGF)-beta1; in 124 infants (85% white, 57% male) to determine any associations between the specific cytokine genotypes and a parental history of allergic rhinitis and/or asthma. The authors demonstrated that the frequencies of TNF-α and TGF-beta1 phenotypes were significantly associated with parental histories of AR and asthma, respectively; with no associations between IFN-gamma, IL-6, and IL-10 genotypes and any of the outcome parameters. Similarly, Minhas and colleagues [29] have shown that the frequency of the G-308A polymorphism in the promoter region of the TNF-α gene was significantly higher in AR patients compared with unrelated healthy control subjects in the Pakistani population. Moreover, assessment of genotype distribution demonstrated that the TNF2 homozygous and TNF1/2 heterozygous genotypes were significantly more common in AR patients compared with control subjects, whereas the normal TNF1 homozygous genotype was more frequent in controls. In a more recent study, Krasznai [30] and colleagues investigated the influence of TNF-α gene238A and-308G polymorphisms in Hungarian AR patients and healthy controls, and showed that in the AR patients these polymorphisms were associated with more pronounced clinical symptoms, higher cytokine and IgE levels, and low PNIF values. Indeed, another recent study has indicated that the -308G polymorphism in the TNF-α gene may also be associated with nasal polyposis [31].

Although there is little information on the role of MRPL4 in the aetiology of AR, this molecule has been increasingly investigated because it has been reported to be a downstream target of hypoxia-inducible factor-1α (HIF-1α) [32], which is critical in immune and inflammatory pathways, and the inhibition of which has been shown to attenuate antigen-induced airway inflammation and hyperresponsiveness in animals [33]. Moreover, the gene encoding MRPL4 is located in close proximity to the ICAM-1 gene on chromosome location 19p13.2. Indeed, one recent study employing a genome-wide association study (GWAS) approach in an ethnic Chinese population in Singapore has indicated that MRPL4 SNP rs8111930 was significantly associated with an AR outcome in this population [2]. In accordance with these findings, here we demonstrated that another locus (rs11668618_CT genotype) of the MRPL4 gene may also be associated with AR risk in the Chinese Han population.

As AR is a polygenic inheritance disease, we have studied the gene polymorphisms associated with the ICAM-1 pathway and its upstream regulatory factors NF-κB and TNF-α, to also investigate any synergistic effects between these polymorphisms and susceptibility to AR. Using MDR software testing, our results showed that the selected sets of polymorphisms had no synergistic effect, even between MRPL4 and ICAM-1 which are located closely on the same chromosomal location. However, this conclusion is limited to present research in the Han Chinese population, and needs to be confirmed in subjects of other ethnicities. However, after adjusting for age and gender, we found that age was the best factor predicting AR risk in all four models of MDR analysis. Although we could not find a synergistic relationship among these variables when we applied the interaction dendrogram and graph, one study from Taiwan showed age and gender to be contributing differentially to different AR subtypes [34]. The risk of developing all atopic diseases is complex and the temporal pattern described in the atopic march [35], [36], [37] may not be a simple progression from atopic dermatitis to AR to asthma, because the development of these diseases is strongly influenced by both genetic and environmental factors. Therefore, age might be an important factor in the atopic march [38], although more research is necessary in this field to confirm the correlations between age and AR.

Last but not least, we would like to acknowledge that the number of the subjects in present study is not as substantial as can be found in other well-financed, multi-center GWAS studies, we nevertheless submit that this is a rather well-characterized population, all have been well-phenotyped according to demographic factors as well as allergen related tests, and are all ethnic Chinese in origin, ensuring a certain homogeneity to the results, which gives this population significant value nonetheless. Additionally, the statistical power of AR vs. control was 95.01%, indicating a relative ideal study sample size.

In summary, present study is the first one to investigate the interactive roles of polymorphisms in the genes coding for ICAM-1 and its upstream regulatory factors NF-κB and TNF-α, as well as MRPL4, a gene located in close vicinity of the ICAM-1 gene. Furthermore, here we investigated associations between these polymorphisms and the risk for developing AR. Our study has suggested that specific TNF-α and MRPL4 gene polymorphisms may be associated with AR susceptibility in a Han Chinese cohort, although these need to be confirmed in additional larger studies including replication in a second cohort as well as the related functional research.
